# Rethinking LPCAT3 roles in human disease: broadening perspectives beyond ferroptosis

**DOI:** 10.1038/s41419-026-08893-w

**Published:** 2026-07-03

**Authors:** Zihan Lin, Qian Jiao, Siqi Liu, Xixun Du, Yu Du, Ying Wang, Mengru Liu

**Affiliations:** 1 Department of Physiology, Shandong Key Laboratory of Pathogenesis and Prevention of Brain Diseases, State Key Disciplines: Physiology, School of Basic Medicine, Qingdao Medical College, Qingdao University, Qingdao, China; 2 Key Laboratory of Neuropharmacology and Translational Medicine of Zhejiang Province, College of Pharmaceutical Science, Zhejiang Chinese Medical University, Hangzhou, China

**Keywords:** Physiology, Cell death

## Abstract

Lysophosphatidylcholine acyltransferase 3 (LPCAT3), a membrane-bound O-acyltransferase, is well known for enriching phospholipids (PLs) with polyunsaturated fatty acids (PUFAs) and thereby driving ferroptosis. However, accumulating evidence indicates that LPCAT3 is not merely a ferroptosis effector but a broader integrator of non-ferroptotic functions, such as autophagy, endoplasmic reticulum homeostasis, and inflammatory signaling. Herein, we provide an overview of LPCAT3 functions that explicitly extend beyond ferroptosis. We first provide a comprehensive review of the structural and enzymatic characteristics of LPCAT3, along with its diverse regulatory mechanisms of expression. Subsequently, we summarize how LPCAT3-mediated PL remodeling modulates membrane biophysical properties and further elucidate the downstream effects of this remodeling on the regulation of autophagy, endoplasmic reticulum homeostasis, and inflammatory signaling pathways. Finally, we systematically review the pivotal roles of LPCAT3 in the pathogenesis of multiple human diseases, including neurodegenerative diseases, stroke, atherosclerosis (AS), diabetes mellitus, obesity (OB), non-alcoholic fatty liver disease (NAFLD), and cancer. This review aims to elucidate the functions of LPCAT3 beyond its role in regulating ferroptosis and may provide new insights into its involvement in human diseases.

## Facts

1. LPCAT3, a key phospholipid remodeling enzyme, mediates remodeling to modulate the biophysical properties of cell membranes.
2. LPCAT3 is regulated transcriptionally and epigenetically.
3. LPCAT3 is involved in regulating ferroptosis, autophagy, endoplasmic reticulum stress, and inflammatory signaling pathways.
4. LPCAT3 plays a pivotal role in the pathogenesis of multiple human diseases, including neurodegenerative diseases, stroke, atherosclerosis, diabetes mellitus, obesity, non-alcoholic fatty liver disease, and malignant tumors, while its role in specific diseases remains controversial.

## Introduction

Phospholipids (PLs) form the main building blocks of cell membranes. The PL bilayer separates the intracellular and extracellular environments, establishes specialized membrane domains, safeguards cellular homeostasis, and facilitates diverse biological processes [1–3]. In mammalian cells, the major structural lipids in PLs membranes are glycerophospholipids such as phosphatidylcholine (PC), phosphatidylethanolamine (PE), phosphatidylserine (PS), phosphatidylinositol (PI), and phosphatidic acid (PA) [4]. These PLs are synthesized de novo through the Kennedy pathway and subsequently remodeled via the Lands cycle, a process involving sequential deacylation and reacylation reactions [5, 6]. The lysophosphatidylcholine acyltransferase (LPCAT) family catalyzes the reacylation step of the Lands cycle [7]. Among the LPCAT family members, LPCAT3 has emerged as a molecule of particular interest owing to its broad tissue distribution and its pivotal role in ferroptosis [8–17].

Ferroptosis is an iron-dependent form of cell death induced by membrane lipid peroxidation, whose dysregulation is associated with the pathogenesis of numerous human diseases [18–21]. LPCAT3 modulates ferroptosis by regulating the incorporation of polyunsaturated fatty acids (PUFAs) into PLs and has recently been shown to be involved in other non-ferroptosis functions, such as autophagy, endoplasmic reticulum (ER) stress, and inflammation, underscoring its multifaceted intracellular functions [11, 15, 22–32]. Furthermore, the roles of LPCAT3 in various human diseases are increasingly being elucidated in neurodegenerative diseases, stroke, atherosclerosis (AS), diabetes mellitus (DM), obesity (OB), non-alcoholic fatty liver disease (NAFLD), and cancer [8, 33–37].

In this review, we systematically summarize the structure and functions of LPCAT3. We also provide an overview of how LPCAT3 participates not only in ferroptosis, but also in autophagy, inflammation, and ER stress. Furthermore, we synthesize current evidence on the pathological roles of LPCAT3 across diverse human diseases, including neurodegenerative diseases, stroke, metabolic diseases, and cancer. Collectively, this review aims to clarify the broader biological repertoire of LPCAT3 beyond ferroptosis regulation and to offer new insights into its contributions to human disease pathogenesis.

## Structure and function of LPCAT3

### Structural features of LPCAT3

**Fig. 1:**
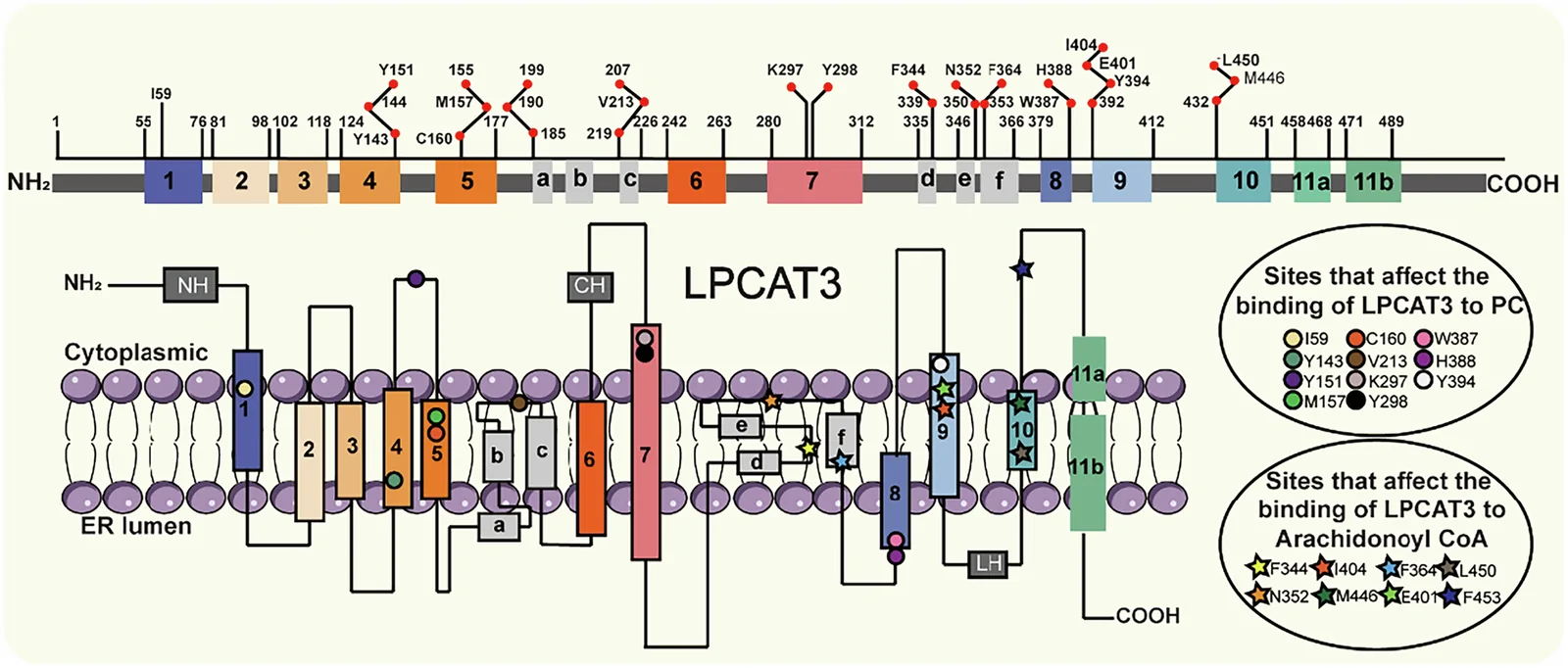
The fundamental structure of LPCAT3. LPCAT3 consists of 502 amino acids and has seventeen domains, including 11 long transmembrane helices and 6 short membrane-embedded helices. Among these helices, helices 1, 7, 9, 10, and 11a span the ER membrane on the cytosolic side, while helices 2–6, 8, 11b, and the six short helices are oriented toward the luminal side of the ER membrane. It also marked the catalytic binding sites of LPCAT3.

LPCAT3 was first cloned by Professor Takao Shimizu’s group in 2008 [38]. LPCAT3 is located on the ER membrane, consists of 502 amino acids and contains 17 structural domains, including 11 long transmembrane (TM) helices and 6 short helices. TM1, TM7, TM9, TM10, and TM11a traverse the ER membrane near the cytosolic side, while TM2-6, TM8, TM11b, and the 6 short helices are oriented toward the luminal side of the ER membrane. Cryo‑EM and computational modeling reveal that LPCAT3 adopts a bell‑shaped architecture, with its two lobes converging at the cytosolic surface to form a large T‑shaped cavity that opens toward the ER lumen. This central cavity contains a vertical tunnel, which serves as the entry site for acyl donors, such as arachidonoyl‑CoA (AA-CoA), and a horizontal channel near the cytosolic face for lysophosphatidylcholine (LPC) entry. A small side pocket connects these tunnels, collectively forming the catalytic chamber decorated with circular loop structures. Structural snapshots of the substrate‑bound and transition states have clarified determinants of substrate specificity and acyltransferase activity. LPCAT3 mutations at F344, N352, E401, I404, M446, L450, or F453 markedly reduce PUFA‑specific activity, with the E401 mutation abolishing enzyme function entirely. Mutations at Y143, Y151, M157, C160, Y298, W387, H388, and Y394 diminish LPC binding, whereas I59 or K297 mutations enhance catalytic efficiency [39] (Fig. 1).

### Physiological functions of LPCAT3

**Fig. 2:**
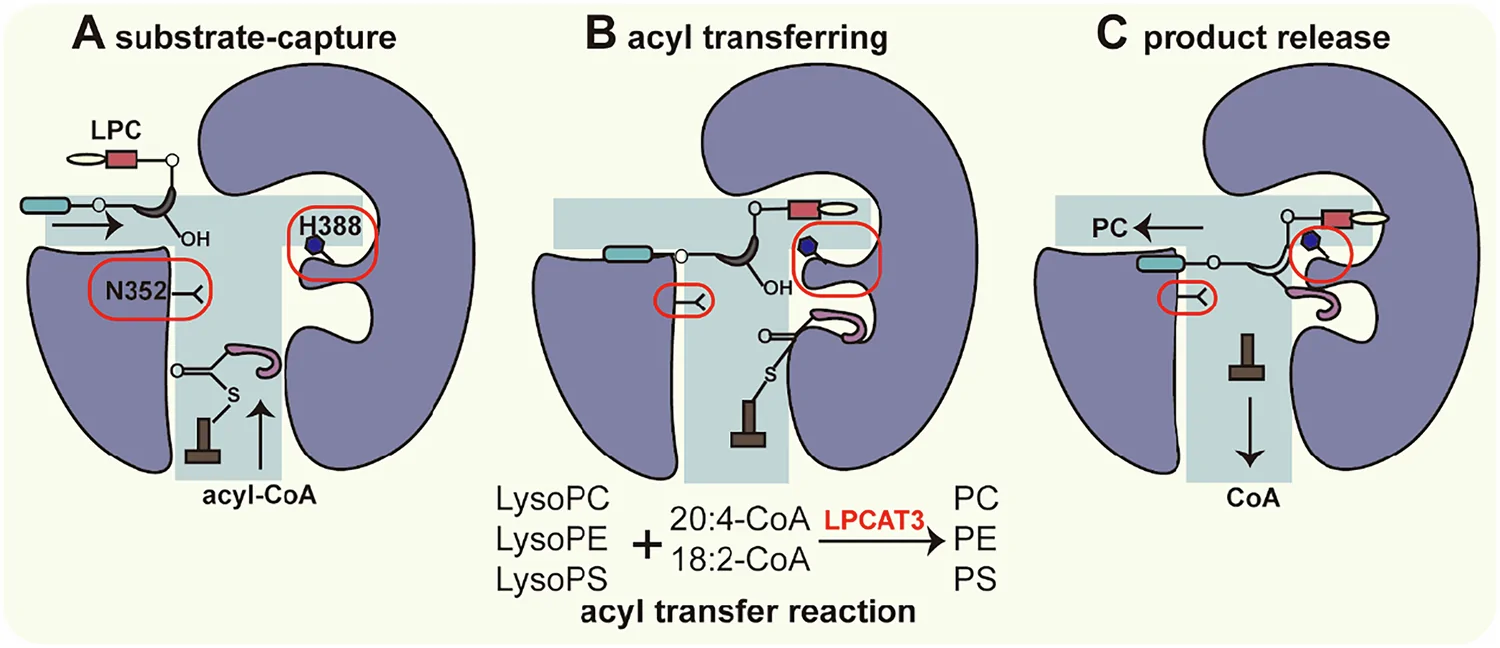
The acyl-transfer reaction of LPCAT3. **A**–**C** Using LPC and AA-CoA as representative substrates, the acyl-transfer reaction catalyzed by LPCAT3 proceeds through three sequential steps, including substrate capture, acyl transfer, and product release.

LPCAT3 functions as a master regulator of membrane PLs composition, exerting its effects through a hierarchical regulatory cascade that ultimately shapes diverse cellular fates. At the molecular level, LPCAT3 preferentially reacylates LPC at the sn-2 position with long chain PUFAs, such as arachidonic acid (AA), eicosapentaenoic acid (EPA), docosahexaenoic acid (DHA), linoleic acid (LA), and α-linolenic acid (ALA), thereby remodeling the biophysical and signaling landscape of cellular membranes [5, 6]. Using LPC and AA-CoA as representative substrates, the acyl-transfer reaction of LPCAT3 progresses through three sequential steps including substrate capture, acyl transfer, and product release (Fig. 2). Therefore, LPCAT3-regulated PLs remodeling enhances membrane flexibility, fluidity, and curvature, while reducing lipid packing density and rigidity by incorporating PUFAs acyl chains containing multiple double bonds [7, 39–42].

Moreover, these changes in membrane biophysical properties, in turn, influence biased signal transduction. For example, elevated membrane fluidity facilitates the lateral mobility and conformational stability of transmembrane proteins, including receptors, transporters, and ion channels. This modulation also promotes clustering and activation of surface receptors, such as G protein coupled receptors (GPCRs), thereby amplifying downstream membrane-associated signaling [43–46].

Furthermore, these membrane and signaling alterations may ultimately bias cell fate decisions, including ferroptosis, autophagy, ER stress and inflammation. For instance, the liberated PUFAs, particularly AA, provide essential precursors for eicosanoid synthesis (prostaglandins and leukotrienes), positioning LPCAT3 as a key node in inflammatory signaling networks [31, 47, 48]. In addition, enrichment of membranes with PUFA-containing PLs renders cells more susceptible to lipid peroxidation, thereby modulating the threshold for ferroptosis [49].

## The expression and regulation of LPCAT3

LPCAT3 is broadly expressed across human tissues but shows marked tissue specificity. Under physiological conditions, its expression is relatively high in the liver, intestine, lung, appendix, placenta, and kidney, and lower in the brain, bone marrow, heart, spleen, and skin. As reported, the level of LPCAT3 is significantly downregulated in AS and NAFLD [34, 50]. Conversely, LPCAT3 is upregulated in stroke, hyperuricemia, and chronic prostatitis [8, 51–53]. However, LPCAT3 remains controversial in Alzheimer’s disease (AD), DM, OB, acute myeloid leukemia, and cancer [9, 14–16, 27, 30, 33, 35, 54–71].

**Fig. 3:**
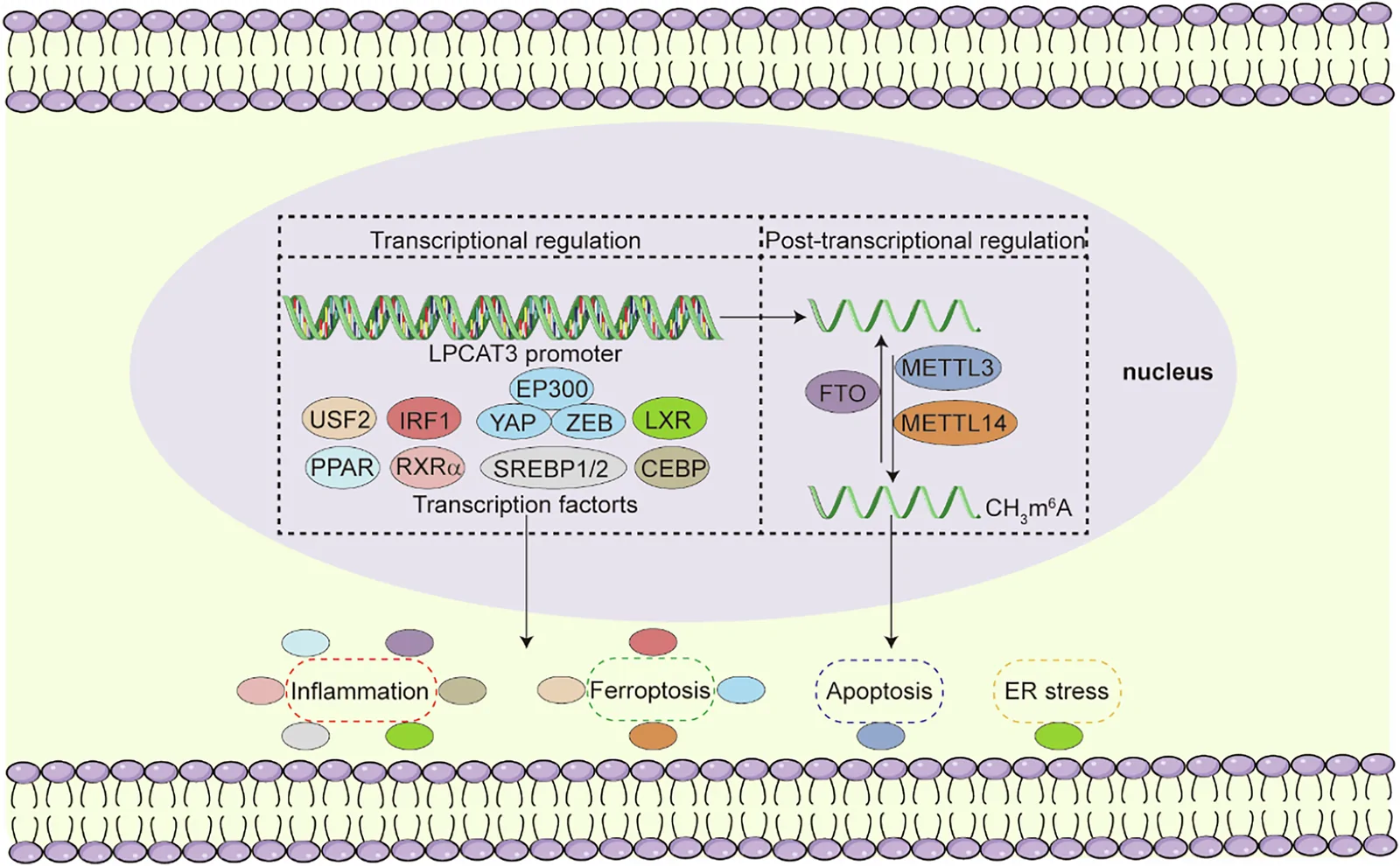
Transcriptional and epigenetic regulation of LPCAT3. The expression of LPCAT3 is regulated by multiple transcription factors, including LXR, USF2, EP300, SREBP1/2, RXRα, PPAR, CEBP and IRF1. Additionally, LPCAT3 is modulated via epigenetic mechanisms. METTL3, FTO, and METTL14 can regulate the m⁶A methylation of LPCAT3 mRNA.

Intriguingly, LPCAT3 is regulated by transcription modification (Fig. 3). Liver X receptor (LXR) is an important transcription factor that regulates LPCAT3 expression. In the liver, LXR-driven LPCAT3 suppresses ER stress and attenuates steatosis, inflammation, and hepatocellular damage [32]. In dorsal root ganglion sensory neurons, LXR-dependent LPCAT3 alleviates ER stress by restoring lipid homeostasis [72]. In human aortic endothelial cells, LXR-induced LPCAT3 remodels membrane lipid composition and regulates the release of lipid mediators [73]. In addition, other transcription factors, such as Upstream Stimulatory Factor 2 (USF2), Yes-associated protein (YAP)/Zinc finger E-box-binding homeobox (ZEB)/E1A-binding protein p300 (EP300) complex, Interferon Regulatory Factor 1 (IRF1), Sterol Regulatory Element-Binding Protein 1 (SREBP1), Retinoid X receptor α (RXRα) and Peroxisome Proliferator-Activated Receptor α/γ (PPAR-α/γ) also modulate LPCAT3. USF2 induces LPCAT3 transcription and promotes ferroptosis, thereby exacerbating sepsis-induced acute kidney injury [17]. In lung adenocarcinoma, YAP/ZEB/EP300 complex increases LPCAT3 transcription levels, thereby rendering cells more susceptible to ferroptosis [14]. In melanoma and lung cancer cells, IRF1 transcriptionally upregulates LPCAT3, thereby promoting ferroptosis and positioning LPCAT3 as a potential predictive biomarker [16]. In HEK 293T cells, nine transcription factors, including LXRα, RXRα, PPARα, PPARγ, and SREBP1, all negatively regulate the expression of LPCAT3, among them, SREBP1 exhibits the most potent regulatory effect [27].

Notably, epigenetic regulation also contributes to LPCAT3 expression (Fig. 3). In repeated UV-induced skin cells, fat mass and OB-associated protein (FTO) depletion elevates LPCAT3 N⁶-methyladenosine (m^6^A) modification, leading to reduced protein expression and aggravated apoptosis [74]. In acute kidney injury, methyltransferase-like 14 (METTL14) depletion reduces LPCAT3 m^6^A methylation, mRNA stability, and expression, thereby inhibiting renal tubular epithelial cell ferroptosis [75]. Methyltransferase-like 3 (METTL3) negatively regulates LPCAT3 by reducing its mRNA stability and abundance via m^6^A methylation [76].

Notably, the functional output of LPCAT3 is shaped not only by its expression level, but also by multiple additional determinants, including its intrinsic enzymatic properties, the availability of intracellular fatty acyl-CoA substrates, functional coupling with ACSL4, its subcellular distribution, and competitive or compensatory interactions with other PLs-remodeling enzymes, such as LPCAT1, LPCAT2 and members of the MBOAT family. For instance, LPCAT3 exhibits substrate selectivity, preferentially utilizing PUFAs rather than saturated fatty acyl-CoAs as acyl donors. Moreover, LPCAT3 and ACSL4 have been reported to interact at the protein level and to jointly influence cellular sensitivity to ferroptosis [14, 76]. Thus, the function of LPCAT3 is best interpreted in a context-dependent manner, with its ultimate effects determined by the convergence of multiple regulatory mechanisms that govern its activity and downstream membrane remodeling outcomes.

## The multifaceted roles of LPCAT3 in cellular homeostasis

**Fig. 4:**
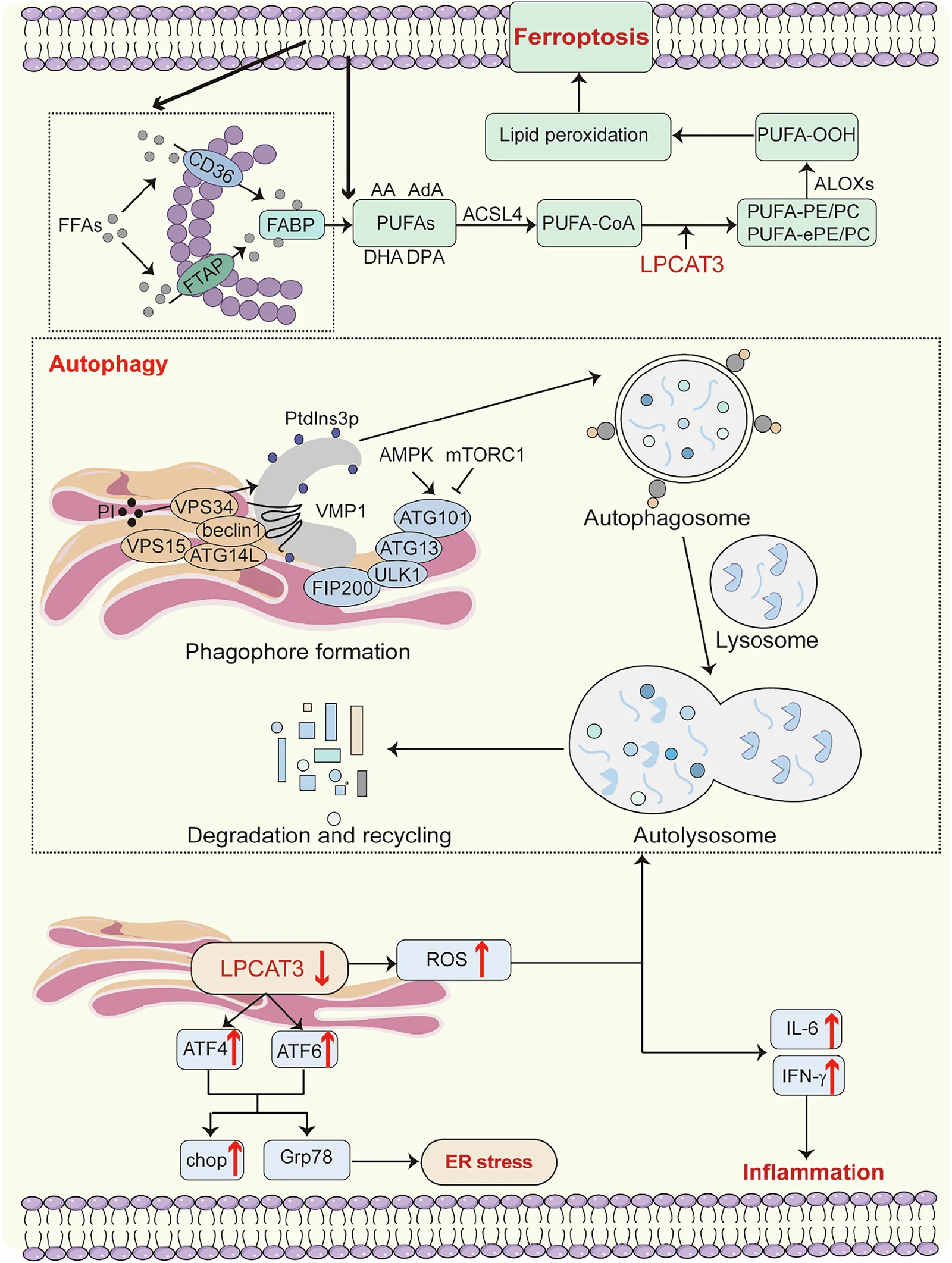
The multifaceted roles of LPCAT3 in cellular homeostasis. LPCAT3 is involved in the processes of ferroptosis, autophagy, ER stress, and inflammation, so it has profound significance in maintaining cellular homeostasis. During the process of ferroptosis, after free fatty acids enter the cell through transporters such as CD36 and FATP, they are transported to specific organelles via FABP. PUFAs are the main substrates for lipid peroxidation. Under the catalysis of a series of enzymes such as LPCAT3, they participate in the synthesis of PLs, and these PLs containing PUFAs are prone to peroxidation, which in turn induces ferroptosis. LPCAT3 is highly expressed on the ER membrane. The reduction of LPCAT3 will affect the changes of downstream signaling pathways, resulting in ER stress. LPCAT3 induces ROS, thereby triggering autophagy and inflammation.

As previously mentioned, LPCAT3 precisely regulates the PLs composition of cell membranes and organelle membranes, especially ER, through its unique acyltransferase activity, thus playing an indispensable role in maintaining normal physiological functions and membrane homeostasis. This section will overview the multifaceted roles of LPCAT3, summarizing its pivotal contributions to four interrelated determinants of cellular homeostasis, including its well-established pro-ferroptotic function, its complex interplay with autophagic flux, its potentiation of inflammatory signaling, and its crucial role in modulating ER stress. Unraveling these diverse functions is essential for comprehending how LPCAT3 integrates metabolic cues to ultimately dictate cell fate in health and disease (Fig. 4).

### LPCAT3 and ferroptosis

#### Ferroptosis

Ferroptosis was first reported by Stockwell’s lab in 2012, it is a kind of cell death which is caused by iron-dependent lipid peroxidation [77]. Morphologically, ferroptotic cells display enlarged cell bodies, swollen organelles, ruptured plasma membranes, and condensed mitochondria with diminished or absent cristae [19]. Lipid peroxides accumulate predominantly through the oxidation of PUFAs moieties within PLs, thereby driving the execution of ferroptosis. Two enzymes, acyl-CoA synthetase long-chain family member 4 (ACSL4) and LPCAT3, are indispensable for the generation of oxidation-susceptible lipid species [78]. Acting in concert through the Lands cycle, they esterify PUFA-derived acyl chains, including arachidonic acid (AA) and adrenic acid (AdA), into membrane PLs. These PUFA-enriched PLs are subsequently oxidized by lipoxygenases (LOXs), giving rise to reactive lipid peroxides that ultimately trigger ferroptosis.

#### Relationship between LPCAT3 and ferroptosis

Accumulating evidence indicates that LPCAT3 modulates ferroptotic susceptibility in a variety of diseases, including ischemic stroke, acute kidney injury, subarachnoid hemorrhage, rabies infection, and multiple types of cancer [8, 13, 75, 79].

In rat subarachnoid hemorrhage, LPCAT3 is elevated and enhances neuronal ferroptosis and early brain injury, whereas LPCAT3 knockdown reduces neuronal death and ameliorates behavioral and cognitive deficits [13]. In acute kidney injury, the RNA methyltransferase METTL14 amplifies ferroptosis through m⁶A-dependent stabilization of LPCAT3 mRNA, leading to higher LPCAT3 protein levels [75]. In rabies virus infection, the natural compound Z-ligustilide, derived from *Ligusticum chuanxiong*, hinders viral reproduction by stimulating the ACSL4/LPCAT3 pathway, thus causing ferroptosis [79]. In lung adenocarcinoma, LPCAT3 overexpression heightens cellular susceptibility to ferroptosis by promoting lipid peroxidation [14]. In severe acute pancreatitis, ACSL4/LPCAT3/arachidonate 15-lipoxygenase (ALOX15) axis further aggravates pancreatic injury [11].

However, gastrodin mitigates neuronal injury and ameliorates neurological dysfunction in ischemic stroke by suppressing the ACSL4/LPCAT3 axis, thereby reducing iron accumulation and lipid peroxidation, preserving mitochondrial ultrastructure and function, and ultimately restraining ferroptosis [8].

Therefore, under the pathological conditions of lung adenocarcinoma and other tumors, LPCAT3 mainly exerts a ferroptotic effect and participates in regulating the sensitivity of tumor cells to ferroptosis. In specific stress/cell protection models, the use of LPCAT3 inhibitors can significantly alleviate ferroptosis damage and protect cells. This bidirectional difference reflects the complexity and dynamic equilibrium of the ferroptosis regulatory network under disease conditions.

### LPCAT3 and autophagy

#### Autophagy

Autophagy is a conserved homeostatic process that degrades cytoplasmic cargos, such as damaged organelles, misfolded proteins, and lipid droplets in lysosomes. It can be divided into three types, macroautophagy, microautophagy, and chaperone-mediated autophagy. Among them, macroautophagy has been the most extensively studied and is hereafter referred to as autophagy. It includes six main steps, induction and initiation, nucleation, membrane elongation and expansion, maturation and closure, autophagosome-lysosome fusion, and lysosomal degradation of cargo [80, 81].

During initiation, echanistic target of rapamycin (mTOR) activity suppresses autophagy, whereas mTOR inhibits the Unc-51-like autophagy-activating kinase 1/2 complex, which recruits the PI3K complex to generate phosphatidylinositol 3-phosphate (PI3P). PI3P then recruits effector proteins and drives phagophore expansion. Membrane elongation is dependent upon the Autophagy-related gene 5-Autophagy-related Gene 12 conjugation system and the Microtubule-associated proteins 1 A/1B light chain 3 (LC3) lipidation cascade, while closure requires fusion proteins such as Syntaxin 17 and Vesicle-associated membrane protein 8 (VAMP8). Autolysosomes are formed via the fusion of mature autophagosomes and lysosomes, a process mediated by Lysosome-associated membrane protein 1/2 (LAMP1/2), the Soluble N-ethylmaleimide-sensitive factor attachment protein receptor complex such as Syntaxin 17-VAMP8-Synaptosomal-associated protein 29, and Ras-Related in Brain GTPases (Rab GTPases), such as Rab7 and Rab21 [46, 82]. Hydrolytic enzymes within lysosomes subsequently degrade the sequestered material.

#### Relationship between LPCAT3 and autophagy

It has been reported that LPCAT3 inhibits autophagy activation. In NASH models, LPCAT3 deficiency has been associated with enhanced autophagic flux, suggesting that LPCAT3 might normally suppress autophagy. Mechanistically, liver-specific LPCAT3 knockout mice show markedly elevated hepatic triglycerides, free fatty acids, and cholesterol, which are further aggravated by a NASH diet. Thus, excess lipids directly injure mitochondria and the ER, and the resulting accumulation of dysfunctional organelles provides an additional trigger for autophagy activation [24, 83]. In addition, LPCAT3 overexpression promotes lipid droplet fusion via nuclear receptor signaling, as evidenced in *C. elegans* where Abnormal Dauer Formation 12 upregulates the homolog MBOA-6 (the *C. elegans* homolog of mammalian LPCAT3) and desaturases (Fatty Acid Desaturase 1–7) to enhance membrane fluidity and drive thermosensitive fusion [84]. Given that the size of autophagosomes is limited, the LPCAT3-mediated expansion of lipid droplets may thereby compromise lipophagy, a form of selective autophagy that specifically targets lipid droplets, thereby possibly promoting hepatocellular lipid accumulation and potentially contributing to the progression of NAFLD.

### LPCAT3 regulate ER stress

The ER is a multifunctional organelle that integrates protein synthesis, folding and quality control with Ca²⁺ storage/signaling and lipid, steroid, and cholesterol biosynthesis. However, extracellular stress, including glucose deprivation, amino acid deficiency, impaired calcium homeostasis, and oxidative stress, can disrupt ER redox homeostasis and compromise its proteostatic capacity. The ensuing collapse of the ER folding environment drives the accumulation of unfolded or misfolded proteins within the ER lumen, culminating in ER stress [85].

A series of studies have revealed that LPCAT3 regulates ER stress in numerous diseases. LPCAT3 inhibition disrupts ER homeostasis by inducing a PLs imbalance that promotes excessive membrane saturation and ER stress. This stress is further amplified through altered ER membrane microdomains that activate cellular Sarcoma tyrosine kinase (c-Src) kinase, driving subsequent inflammatory injury [29]. Upregulation of LPCAT3 mitigates alcohol-induced ER stress, thereby alleviating inflammation and hepatic steatosis [32]. In tumors, elevated LPCAT3 can facilitate the coupling of unsaturated lipid species with PC, thereby attenuating ER stress and supporting tumor cell adaptation [86]. Thus, loss or functional impairment of LPCAT3 triggers ER stress. The reduction in LPCAT3 stiffens the ER membrane, disrupting its protein-folding capacity and resulting in proteostasis failure that directly instigates ER stress. Notably, ER stress can trigger a compensatory feedback loop in which activation of the protein kinase RNA-like ER kinase-eukaryotic initiation factor 2α-activating transcription factor 4 (ATF4) pathway upregulates LPCAT3, thereby promoting restoration of membrane homeostasis and attenuation of ER stress [32].

### LPCAT3 and inflammation

LPCAT3 also plays a multifaceted role in inflammation, functioning not as a simple pro- or anti‑inflammatory factor but as a finely tuned lipid-metabolic regulator that determines the availability of inflammatory precursors. Its primary substrate, AA, is a central bioactive lipid that fuels the biosynthesis of eicosanoids which mediate classical inflammatory manifestations including fever, pain, vasodilation, and leukocyte recruitment. Under basal conditions, free AA is tightly sequestered in membrane PLs to prevent uncontrolled inflammatory signaling [87]. LPCAT3 acts as an AA-loading enzyme, efficiently re-esterifying free AA into PLs pools to maintain cellular homeostasis [88].

During inflammation, phospholipase A₂ (PLA₂) releases AA from membrane PLs into the cytosol, where it is rapidly converted into inflammatory mediators. The amount of LPCAT3-dependent AA storage thereby influences the magnitude of the inflammatory response. High LPCAT3 increases the reservoir of esterified AA available for mobilization, positioning LPCAT3 as a potential pro-inflammatory amplifier. Conversely, by rapidly clearing cytosolic AA via re-esterification, LPCAT3 contributes to inflammation resolution, reflecting its context-dependent duality [89].

Mice with liver-specific LPCAT3 deficiency spontaneously develop NASH over time, characterized by mitochondrial dysfunction, ER stress, and activation of the JNK pathway [24]. Overexpression of LPCAT3 mitigates hepatic lipid accumulation and inflammation, whereas knockdown exacerbates disease phenotypes [24]. In contrast, in palmitic-/oleic-acid-stimulated cell models, LPCAT3 deficiency alleviates ER stress and inflammation, emphasizing a tissue‑dependent dichotomy [90].

In macrophages, LPCAT3 is tightly regulated by inflammatory stimuli. It may be transiently upregulated during the acute phase to ensure effective immune responses, and subsequently downregulated during the resolution phase. Dysregulation of this cycle could lead to chronic inflammation or immune exhaustion [89]. Given this nuanced control, LPCAT3 becomes an interesting potential therapeutic target for chronic inflammation. Rather than uniform inhibition or activation, precision modulation of LPCAT3 tailored to tissue context may offer a route toward restoring lipid and inflammatory homeostasis.

## The role of LPCAT3 in human disease

**Fig. 5:**
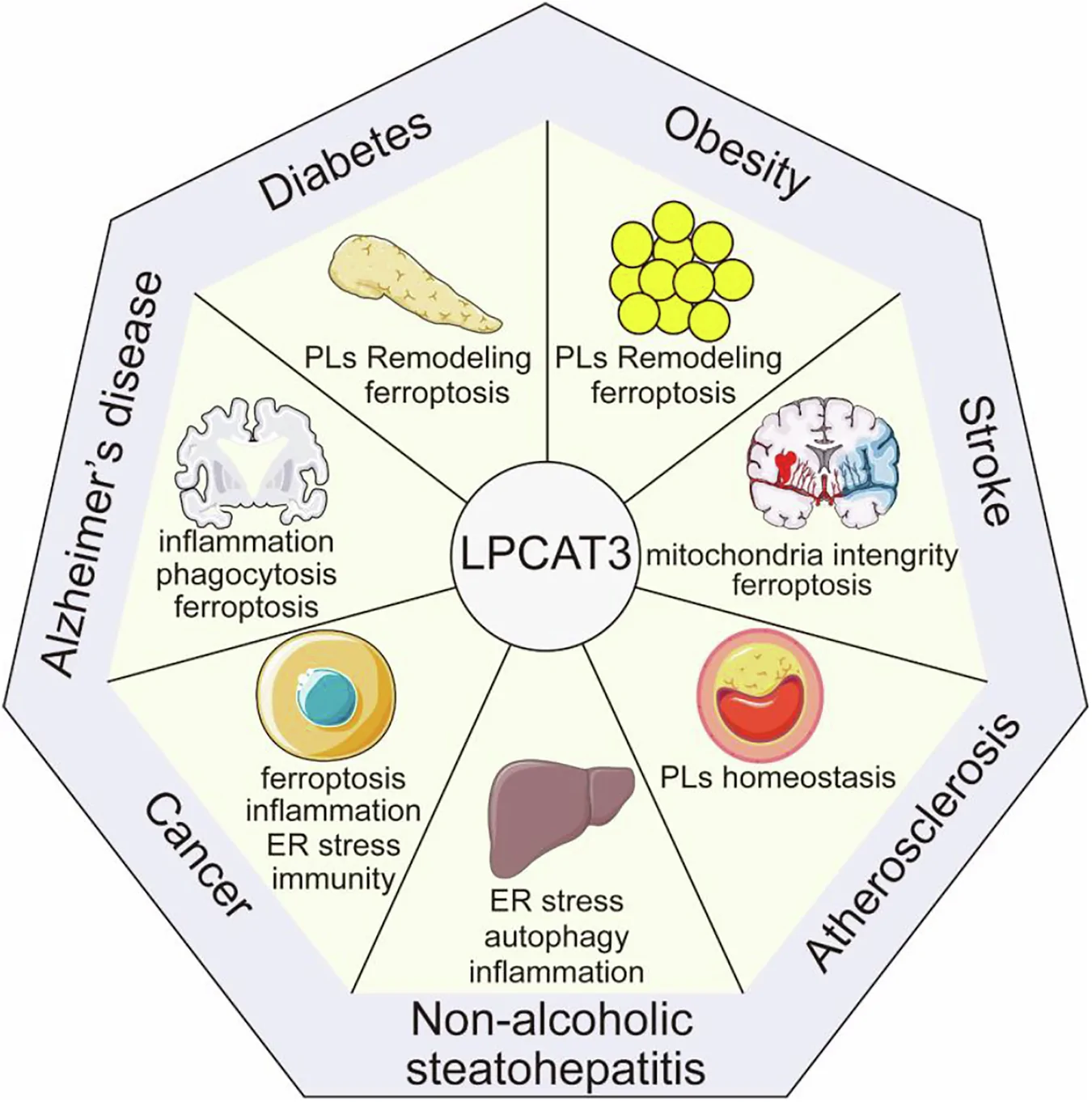
LPCAT3 and human diseases. LPCAT3 plays an important role in human diseases, and its functions may vary depending on the tissue and cell types. Specifically, LPCAT3 can mediate ferroptosis and participate in the pathogenesis of diabetes, obesity, stroke, AD, and cancer. Besides, LPCAT3 can mediate inflammation to affect the pathogenesis of AD and AS. Additionally, LPCAT3 can affect the occurrence and development of cancer and NASH by regulating ER stress.

Emerging evidence reveals the involvement of LPCAT3 in multiple human diseases, including neurodegenerative diseases, stroke, metabolic disorders, and cancer. In this section, we will systematically review the multifaceted functions of LPCAT3 in several human diseases (Fig. 5 and Table 1).

**Table 1:** The roles of LPCAT3 in human diseases.

Disease	Effect	Model	Mechanism	Agents
Alzheimer’s disease	Negative Negative	App^NL-GF^ mice APP/PS1 mice	Phagocytosis [54] Ferroptosis [55]	Mixture [55]
	Negative		Inflammation [96]	
Stroke	Negative	MCAO/R rat	Ferroptosis [8]	Gastrodin [8]
	Negative	dMCAO mice	Mitochondria integrity [99]	
Atherosclerosis	Positive	macrophages	PL homeostasis [34]	
	No effect	HFD mice [50]		
Diabetes mellitus	Positive	Mice (adenovirus)	Lipoprotein changes [56]	
	Negative	HFD ob/ob mice	PL remodeling [58]	
	Negative	HFD ob/ob mice	Membrane saturation [59]	
	Negative	HSkMCs	PL composition [60]	
	Negative	HFD ob/ob mice	PL remodeling [57]	
Obesity	Negative	PexRAP mice	Lipogenesis [35]	
	Positive	HFD mice	PL remodeling [61]	
Non-alcoholic fatty liver disease	Positive	LKO mice	Autophagy [24] Inflammation	Qinggan [32] Diosgenin [119]
	Negative	HFD mice	ER stress [90] inflammation	
Cancer	Positive	Lung adenocarcinoma	Ferroptosis [14]	Mefloquine [16] Nanoparticle [120]
	Positive	Melanoma	Ferroptosis [16]	
	Positive	Renal cancer	ER stress [30]	

### LPCAT3 and AD

AD is a neurodegenerative disease with β-amyloid plaques, neurofibrillary tangles, synaptic loss, neuroinflammation, and region‑specific atrophy [91–94]. Pathogenetic hypotheses encompass protein misfolding toxicity, acetylcholine deficiency, mitochondrial dysfunction, oxidative stress, lipid dysregulation, and ferroptosis.

Dysregulation of AA metabolism and signal transduction is closely associated with nervous system diseases, particularly AD [95]. In the brain, AA is typically remodeled via the deacetylation-reacetylation cycle, in which the reacylation process catalyzed by LPCAT3 may, in part, regulate the levels of free AA and AA-containing PLs [31]. Peroxidation products derived from AA may contribute to the pathogenesis of AD by elevating oxidative stress and neuroinflammation, such as acrolein, 4-hydroxyhexenal, and 4-hydroxydecadienal. The compounds subsequently formed by these products exacerbate neuronal damage and further promote neuroinflammation [96]. In addition, lipid peroxidation can induce ferroptosis in neurons and glial cells. In the brains of AD patients, accumulation of lipid peroxides, as well as Aβ plaques and tau proteinopathies, can be observed [97]. In primary microglia isolated from Amyloid Precursor Protein NL-GF AD mouse models, LPCAT3 deletion reduces oxidative stress and inflammation, enhances phagocytosis, and partially reverses amyloid-dependent neuropathology [54]. Similarly, in Amyloid Precursor Protein/Presenilin 1 (APP/PS1) transgenic mice, downregulation of LPCAT3 suppresses lipid peroxidation-linked ferroptosis and significantly improves cognitive performance [55].

In summary, LPCAT3 may contribute to AD pathogenesis by enhancing oxidative stress, neuroinflammation and ferroptosis, while alleviating amyloid-dependent neuropathology.

### LPCAT3 and stroke

Stroke, including ischemic and hemorrhagic types, continues to be one of the main reasons for deaths and disabilities among adults all over the world. Neuron death is a central occurrence in stroke [98].

Dysregulation of the ACSL4/LPCAT3 and xCT/GPX4 pathways is key steps for ferroptosis in stroke. In the experimental rat model of middle cerebral artery occlusion/reperfusion and the PC12 cell model of oxygen-glucose deprivation/reoxygenation injury, disruption of the xCT/GPX4 axis, coupled with activation of the ACSL4/LPCAT3 axis, promotes ferroptosis and thereby contributes to neuronal injury [8]. Besides, LPCAT3 inhibition enhances mitochondrial integrity, thereby alleviating neuronal injury [99].

Taken together, LPCAT3 exacerbates neuronal injury primarily by compromising mitochondrial integrity and promoting ferroptosis in stroke.

### LPCAT3 and AS

AS is a persistent pathological disorder that causes changes in the structure and function of the arterial intima-media complex. The pathophysiological cascade starts when ectopic lipids accumulate in the subendothelial intima, followed by extracellular matrix remodeling, intraplaque microhemorrhage, thrombosis, and ultimately the formation of a fibroatheroma [100, 101].

Elevated LPCAT3 expression has been observed in atherosclerotic lesions and correlates positively with disease severity [102–104]. Mechanistically, LPCAT3 loss may exacerbate disease progression by impairing lipid processing and cholesterol export in AS. It has been reported that inhibition of LPCAT3 may confer short-term metabolic benefits, which is manifested in that inhibition of LPCAT3 significantly reduces polyunsaturated PC in the intestinal plasma membrane, and decreases the levels of Niemann-Pick C1-Like 1, Cluster of Differentiation 36 (CD36), ATP-binding cassette subfamily A member 1, and ATP-binding cassette subfamily G member 8 on the membrane, thereby significantly reducing lipid absorption [105]. In addition, LPCAT3 regulates inflammation in AS. Besides, macrophage deficiency of LPCAT3 reduces the proportion of PUFA-PC within lipid rafts, leading to c-Src phosphorylation and upregulation of Toll-like receptor 4 (TLR4) [106]. This activates mitogen-activated protein kinase and nuclear factor kappa-light-chain-enhancer of activated B cells signaling pathways, amplifying cytokine production and fostering a pro-inflammatory phenotype that destabilizes plaques. In mice with hematopoietic cell reconstitution deficient in LPCAT3, macrophage infiltration is significantly increased in atherosclerotic lesions of the aortic valve and aortic arch. The infiltrated macrophages remain in a foam cell state due to defective cholesterol efflux, and are unable to effectively clear lipid deposits, which further promotes the progression of the lesions [34].

Despite these findings, the evidence remains incongruent. It has been reported that bone marrow-specific LPCAT3 knockout exerts negligible effects on macrophage inflammation or ER stress [50]. These discrepancies underscore that key questions remain regarding why LPCAT3 executes distinct functions across different cell types.

Collectively, in AS, LPCAT3 exerts context-dependent effects on lipid metabolism, inflammatory signaling and plaque progression. Although LPCAT3 deficiency may reduce intestinal lipid absorption in the short term, its loss in macrophages disrupts membrane phospholipid remodeling, impairs cholesterol efflux, enhances pro-inflammatory signaling and promotes foam cell persistence, thereby favoring plaque progression and instability. Overall, current evidence suggests that LPCAT3 is neither uniformly protective nor deleterious in AS, but instead modulates disease progression through cell type-specific effects on membrane lipid composition, metabolic flux and inflammatory tone.

### LPCAT3 and DM

DM is a metabolic disorder that has chronic high blood sugar and abnormal insulin signaling. Type 1 DM is caused by immune system wrongly attacks and destroys pancreatic β cells, whereas Type 2 DM results from a mix of insulin resistance and dysfunctional pancreatic β cells [107–109].

LPCAT3 has emerged as a pivotal modulator of insulin sensitivity. LPCAT3 deficiency reduces PUFAs content, increases saturated lipid ratios, and stiffens membranes, thereby impairing insulin actions. In high-fat diet models, acute LPCAT3 deficiency improves insulin sensitivity and alleviates metabolic stress [57, 58], suggesting that reduced LPCAT3 can transiently benefit glucose homeostasis. Intriguingly, hepatic overexpression of LPCAT3 enhances postprandial glucose tolerance, implying its tissue‑specific effects. LPCAT3 may also function as a ferroptosis signal and contribute to β‑cell injury [56]. Since AA serves as a pro‑inflammatory precursor, elevated LPCAT3 increases the PLs reservoir for eicosanoid synthesis, magnifying chronic inflammation, a principal driver of insulin resistance [63]. Furthermore, in diabetic erectile dysfunction, inhibition of ferroptosis restores vascular function [110].

In conclusion, LPCAT3 exerts a dual role in DM. LPCAT3 maintains insulin sensitivity by alleviating cell membrane rigidification, yet it also induces insulin resistance by promoting inflammation and ferroptosis.

### LPCAT3 and OB

OB, a chronic metabolic disorder of multifactorial origin, is clinically defined as abnormal adipose tissue accumulation arising from a persistent imbalance between excessive energy intake and insufficient energy expenditure, when such accumulation elicits measurable impairments to physiological health [111].

In the liver, LPCAT3 facilitates triglyceride export by maintaining an optimal PC/triglyceride ratio necessary for very‑low‑density lipoprotein assembly and secretion. By producing PUFA-rich PC species, LPCAT3 supports very low-density Lipoprotein maturation and prevents intracellular triglyceride accumulation, thus guarding against steatosis. However, excessive LPCAT3 activation may also enrich membranes with AA-containing PC species, fueling inflammatory and fibrotic pathways. LPCAT3 was increased in hyperplastic adipose tissue and mature 3T3-L1 adipocytes [26], which inhibited insulin signaling and glucose tolerance via glycerophospholipid remodeling. Transcriptional regulators including PPARγ, which promotes LPCAT3 expression [61], and LXR, which induces lipogenesis and LPCAT3 upregulation, further link LPCAT3 to metabolic expansion and OB progression. LPCAT3-deficient mice thrive on carbohydrate-rich diets yet fail to tolerate triglyceride-rich regimens, underscoring the enzyme’s necessity for intestinal lipid absorption [34].

In OB, LPCAT3 promotes triglyceride efflux and prevents hepatic steatosis. However, excessive activation of LPCAT3 may trigger inflammatory responses. Therefore, precise and balanced regulation of LPCAT3 is essential.

### LPCAT3 and NAFLD

NAFLD stands as the most widespread chronic liver disease worldwide, including NAFL, NASH, liver cirrhosis, and hepatocellular carcinoma [112]. The advanced stages of NAFLD, especially NASH, are significantly associated with an increased mortality rate [113]. The prevalence of NAFLD worldwide reached 38.0% from 2016 to 2019, an increase of 50.4% compared to 25.3% from 1990 to 2006 [114]. Thus, it’s important to find effective methods and therapeutic targets.

LPCAT3 is involved in the development of NASH. LPCAT3 is greatly reduced in the livers of people with NASH, and this reduction is linked to a higher NAFLD score and more advanced fibrosis stages [24]. In the gene knockout LKO mouse model, overexpression of Lpcat3 can alleviate the inflammation and fibrosis associated with NASH [24]. However, in the high-fat diet (HFD) mouse model, reducing LPCAT3 improved endogenous stress and reduced liver steatosis and inflammation [90]. LXR is a transcription factor that positively regulates LPCAT3. LXR agonists (GW3965) increase the synthesis of polyunsaturated PLs, alleviate ER stress and liver fibrosis in NASH mouse models [115].

The role of LPCAT3 in NASH exhibits a model-dependent controversy. Hepatic LPCAT3 is markedly reduced in human NASH, suggesting that its downregulation may be associated with chronic metabolic stress and disease progression. In LPCAT3 KO mouse models, LPCAT3 deficiency leads to long-term compensatory adaptation and paradoxically alleviates NASH-related pathological changes [24]. By contrast, in high-fat diet-induced mouse models, which represent acute metabolic stress, LPCAT3 downregulation exacerbates pathogenesis, whereas restoring LPCAT3 via overexpression or pharmacological activation ameliorates hepatic lipotoxicity, endoplasmic reticulum stress, and inflammation [90].

These discrepancies arise primarily from the fundamental differences between long-term compensatory reprogramming in genetic models and acute stress-induced injury in dietary models, and are further modulated by species-specific metabolic characteristics, disease stages, and the relative contributions of pathogenic pathways. Therefore, LPCAT3 cannot be simply categorized as “protective” or “detrimental”; instead, it exerts context-dependent dual functions in the maintenance of metabolic homeostasis and the response to metabolic stress.

### LPCAT3 and cancer

Cancer is essentially a disease defined by malignant cellular proliferation, arising from normal cells that evade the body’s homeostatic regulation following successive rounds of genetic-level mutations (proto-oncogene activation and tumor suppressor gene inactivation), concurrent with epigenetic modification alterations and the establishment of immune evasion mechanisms [116]. Cancer has become a major public health issue all over the world. According to surveillance data from the WHO, the annual number of newly diagnosed cancer cases worldwide exceeds 20 million [117].

The expression level of LPCAT3 is significantly increased, and it is usually associated with prognosis, immune infiltration, or serves as a potential therapeutic target. Wide cancer genome analyses (TCGA, GTEx) show that LPCAT3 is elevated in many tumors, such as clear cell renal cell carcinoma, colon adenocarcinoma, acute myeloid leukemia, low-grade glioma, ovarian cancer, and uveal melanoma [30, 64].

It is reported that LPCAT3-induced ferroptosis can alleviate cancer. In lung adenocarcinoma cells, LPCAT3 expression positively correlates with lipid peroxidation, ferroptosis sensitivity, and cell death [14]. In melanoma, the antimalarial drug mefloquine enhances immunotherapy efficacy by activating the IFN-γ and signal transducer and activator of transcription 1/IRF1/LPCAT3 axis, increasing tumor ferroptosis [16]. Particularly in acute myeloid leukemia, LPCAT3 can be a possible predictive biomarker and treatment target [64]. In renal cancer patients, LPCAT3 affects immune infiltration via ER stress regulation, making it a potential prognostic and immunotherapy target [30].

LPCAT3 is closely related to the tumor immune microenvironment. The expression level of LPCAT3 affects the infiltration of immune cells (such as B cells, CD4^+^ T cells, CD8^+^ T cells, macrophages, dendritic cells, etc.), and is positively correlated with the expression of multiple immune checkpoint molecules (such as PDCD1, CTLA4, LAG3, CD274, etc.) [64]. In intestinal tumors, LPCAT3 deficiency greatly increases tumor formation since LPCAT3 deficiency can boost the mitosis of intestinal stem cells via enhancing cholesterol biosynthesis, and ISCs are identified as the origin cells of intestinal tumors [68].

However, in serous ovarian cancer, LPCAT3 is downregulated. Studies have demonstrated that LPCAT3 suppresses the proliferation, migration, and invasion of serous ovarian cancer by inducing ferroptosis in tumor cells via promoting PE-AA synthesis and by inhibiting the EGFR/Akt/mTOR pro-survival signaling pathway through the regulation of AA metabolism [15]. The divergent expression patterns of LPCAT3 across cancer types underscore its context-dependent functions as a central phospholipid-remodeling enzyme within the complex tumor ecosystem. Rather than occurring randomly, these differences are likely dictated by tumor-type specificity, distinct molecular regulatory networks, cellular origin, and microenvironmental context.

LPCAT3 alleviates cancer by enhancing ferroptosis, modulates ER stress to influence immune infiltration, and shapes the tumor immune microenvironment. Additionally, LPCAT3 reduces mitosis of intestinal stem cells by regulating cholesterol biosynthesis, thereby indirectly inhibiting intestinal tumorigenesis.

## Pharmacological modulation of LPCAT3

Accumulating evidence indicates that a subset of pharmacological agents and natural bioactive compounds exert their effects by upregulating LPCAT3 or LPCAT3-associated pathways, thereby promoting lipid peroxidation and ferroptotic vulnerability. For example, Mefloquine has been shown to upregulate LPCAT3 and induce ferroptosis in tumor cells [16]. The plant-derived compound Z-ligustilide activates ferroptosis through the ACSL4–LPCAT3–POR axis while simultaneously suppressing rabies virus replication, thus suggesting a previously unappreciated antiviral application for LPCAT3-centered modulation [79]. Similarly, ginsenoside RK1, a major bioactive constituent of ginseng, induces ferroptosis and attenuates liver fibrosis through activation of the ACSL4/LPCAT3/ALOX5 pathway [9]. In addition, certain nanoparticle-based formulations enhance tumoural reactive oxygen species accumulation by stimulating ACSL4/LPCAT3 signaling, thereby intensifying oxidative stress and ferroptosis [118].

Conversely, another class of interventions appears to confer protection by suppressing LPCAT3 activity or attenuating its downstream signaling, thereby limiting lipid peroxidation and ferroptosis-associated tissue injury. LPCAT3 inhibitors have been reported to rapidly reorganize polyunsaturated phospholipid pools in human cells, mitigating ferroptosis driven by lipid oxidation. Gastrodin suppresses the ACSL4/LPCAT3 pathway, reduces post-stroke ferroptosis, restores mitochondrial membrane potential, and preserves mitochondrial integrity [8]. Qinggan Huoxue Formula lowers LPCAT3 expression and dampens endoplasmic reticulum stress, thereby alleviating alcohol-induced liver injury [32]. In animal models of AD, a standardized combination of epimedium flavonoids, astragaloside IV, and puerarin suppresses LPCAT3 expression and inhibits ferroptotic damage [54]. Diosgenin similarly ameliorates non-alcoholic fatty liver disease through inhibition of the ACSL4/LPCAT3/ALOX15 axis [117]. Curcumin-loaded nanoparticles (Cur-NPs) downregulate LPCAT3, suppress cardiomyocyte ferroptosis, and attenuate myocardial injury [119]. Notably, recent work further suggests that LPCAT3 may function as a procoagulant membrane regulator responsive to aspirin, raising the possibility that it may represent a tractable target for thrombosis risk stratification and intervention [118].

Collectively, most currently identified LPCAT3-targeting compounds appear to act primarily through modulation of ferroptosis, thereby producing antitumour, antifibrotic, or organ-protective effects. Only a limited number of agents, such as Z-ligustilide and aspirin, have thus far demonstrated relatively well-defined ferroptosis-independent functions. However, the field remains largely confined to the preclinical stage, and robust clinical evidence supporting safety, efficacy, and tissue selectivity is still lacking. A major conceptual challenge lies in the pronounced context dependence of LPCAT3 biology: its functional consequences may vary substantially across disease types, disease stages, and cellular environments. Without a precise understanding of this conditionality, therapeutic targeting of LPCAT3 may yield unintended off-target or maladaptive effects. Future efforts should therefore focus on defining the disease-specific phenotypic signatures of LPCAT3 activity, optimizing targeted delivery systems to enhance tissue selectivity, and accelerating translational validation in clinical settings. More broadly, systematic dissection of the interplay between LPCAT3 and other lipid metabolic and membrane-remodeling pathways may reveal additional therapeutic nodes and further expand the clinical potential of this pathway.

## Conclusion and perspectives

LPCAT3, a membrane-bound O-acyltransferase, is a central orchestrator of PLs remodeling that integrates metabolic, inflammatory, and cell-death signals across multiple organ systems. By esterifying PUFAs into membrane PLs, LPCAT3 not only controls ferroptosis susceptibility, but also shapes membrane biophysics, receptor signaling, lipid mediator production, and organelle homeostasis. Thus, LPCAT3 operates as a systems-level regulator in human disease, extending far beyond its canonical role in ferroptosis.

In metabolic and inflammatory diseases, LPCAT3 often acts as a pathogenic enhancer. Its increased has been associated with the aggravation of insulin resistance, the accumulation of inflammatory lipid mediators, and the acceleration of disease progression, including OB, type 2 DM, AS, and AD. Pharmacological or genetic inhibition of LPCAT3 may restore lipid homeostasis and reduce inflammation.

In cancer, the same PUFA-incorporating activity converts LPCAT3 into a disease-dependent vulnerability. By enriching membranes with highly oxidizable PLs, LPCAT3 increases ferroptosis readiness and can sensitize tumor cells to ferroptosis-inducing agents and immunotherapy. High LPCAT3 expression is associated with increased ferroptosis sensitivity in several malignancies, whereas its downregulation can favor survival under oxidative and therapeutic stress. At the same time, LPCAT3-mediated provision of AA for prostaglandin and leukotriene synthesis may foster angiogenesis, immune evasion, and metastatic behavior in specific tumor types. These observations indicate that LPCAT3 can act as both a tumor-suppressive and a tumor-promoting factor based on the type of tissue, the presence of oncogenic factors, and the surrounding environment.

Despite substantial progress, key aspects of LPCAT3 biology remain unresolved. Questions include how transcriptional, epigenetic, and post-translational mechanisms converge to set LPCAT3 in a tissue- and organelle-specific manner; how LPCAT3 differentially engages ferroptosis, autophagy, ER stress responses, and inflammatory signaling in distinct cell types; and why LPCAT3 promotes insulin resistance in hepatocytes yet drives ferroptosis in liver cancer cells.

Besides, future research should aim to develop therapeutic interventions that modulate LPCAT3 with high specificity. In metabolic and inflammatory diseases, organ-specific inhibitors, such as liver-targeted compounds, could restore lipid metabolism without causing systemic effects. In oncology, particularly in drug-resistant tumors, agents that increase or stabilize LPCAT3 could serve as ferroptosis sensitizers and improve the efficacy of existing cytotoxic and immunotherapeutic treatments. The development of such context-specific strategies will be critical for translating the biological roles of LPCAT3 into clinical applications.

## References

[1] Wymann MP, Schneiter R. Lipid signalling in disease. Nat Rev Mol Cell Biol. 2008;9:162–76. 10.1038/nrm2335 18216772

[2] Spector AA, Yorek MA. Membrane lipid composition and cellular function. J Lipid Res. 1985;26:1015–35. 3906008

[3] Van Meer G, Voelker DR, Feigenson GW. Membrane lipids: where they are and how they behave. Nat Rev Mol Cell Biol. 2008;9:112–24. 10.1038/nrm2330 PMC2642958 18216768

[4] Kent C. Eukaryotic phospholipid biosynthesis. Annu Rev Biochem. 1995;64:315–43. 10.1146/annurev.bi.64.070195.001531 7574485

[5] Lands WEM. Metabolism of glycerolipides: a comparison of lecithin and triglyceride synthesis. J Biol Chem. 1958;231:883–8. 13539023

[6] Kennedy EP, Weiss SB. The function of cytidine coenzymes in the biosynthesis of phospholipids. J Biol Chem. 1956;222:193–214. 13366993

[7] Wang B, Tontonoz P. Phospholipid remodeling in physiology and disease. Annu Rev Physiol. 2019;81:165–88. 10.1146/annurev-physiol-020518-114444 PMC7008953 30379616

[8] Gong C, Fu X, Ma Q, He M, Zhu X, Liu L, et al. Gastrodin: modulating the xCT/GPX4 and ACSL4/LPCAT3 pathways to inhibit ferroptosis after ischemic stroke. Phytomed Int J Phytother Phytopharm. 2025;136:156331. 10.1016/j.phymed.2024.156331 39731833

[9] Xia Z, Wang Y, Zhao Q, Deng M, Li C, Zeng Y, et al. Ginsenoside RK1 promotes hepatic stellate cell glycolysis-mediated ferroptosis by activating the HK2/ACSL4/LPCAT3/ALOX5 signaling pathway. J Agric Food Chem. 2025;73:17205–18. 10.1021/acs.jafc.5c00107 40570179

[10] Zhou Y, Wu Y, Zhu R. KLF6 enhances ferroptosis in S-AKI through the NCOA4/ACSL4/LPCAT3 axis. Int Immunopharmacol. 2025;164:115376. 10.1016/j.intimp.2025.115376 40811945

[11] Zhang T, Huang X, Feng S, Shao H. Lactate-dependent HIF1A transcriptional activation exacerbates severe acute pancreatitis through the ACSL4/LPCAT3/ALOX15 pathway induced ferroptosis. J Cell Biochem. 2025;126:e30687. 10.1002/jcb.30687 39676583

[12] Cao N, Zhang F, Yin J, Zhang J, Bian X, Zheng G, et al. LPCAT2 inhibits colorectal cancer progression via the PRMT1/SLC7A11 axis. Oncogene. 2024;43:1714–25. 10.1038/s41388-024-02996-4 PMC11136653 38605214

[13] Hao J, Wang T, Cao C, Li X, Li H, Gao H, et al. LPCAT3 exacerbates early brain injury and ferroptosis after subarachnoid hemorrhage in rats. Brain Res. 2024;1832:148864. 10.1016/j.brainres.2024.148864 38484924

[14] Cui J, Wang Y, Tian X, Miao Y, Ma L, Zhang C, et al. LPCAT3 is transcriptionally regulated by YAP/ZEB/EP300 and collaborates with ACSL4 and YAP to determine ferroptosis sensitivity. Antioxid Redox Signal. 2023;39:491–511. 10.1089/ars.2023.0237 37166352

[15] Wen F, Ling H, Ran R, Li X, Wang H, Liu Q, et al. LPCAT3 regulates the proliferation and metastasis of serous ovarian cancer by modulating arachidonic acid. Transl Oncol. 2025;52:102256. 10.1016/j.tranon.2024.102256 PMC11743812 39733744

[16] Tao Q, Liu N, Wu J, Chen J, Chen X, Peng C. Mefloquine enhances the efficacy of anti-PD-1 immunotherapy via IFN-γ-STAT1-IRF1-LPCAT3-induced ferroptosis in tumors. J Immunother Cancer. 2024;12:e008554. 10.1136/jitc-2023-008554 PMC10936479 38471712

[17] Huang R, Guan Y, Huang W, Shang Y, Xu Y, Li S, et al. USF2 exacerbates sepsis-induced acute kidney injury and ferroptosis through lpcat3-mediated NRF2/HO-1/GPX4 pathway. Shock. 2025;64:97–105. 10.1097/SHK.0000000000002588 40138726

[18] Zeng F, Nijiati S, Tang L, Ye J, Zhou Z, Chen X. Ferroptosis detection: from approaches to applications. Angew Chem Int Ed Engl. 2023;62:e202300379. 10.1002/anie.202300379 36828775

[19] Dixon SJ, Olzmann JA. The cell biology of ferroptosis. Nat Rev Mol Cell Biol. 2024;25:424–42. 10.1038/s41580-024-00703-5 PMC12187608 38366038

[20] Wu X, Li Y, Zhang S, Zhou X. Ferroptosis as a novel therapeutic target for cardiovascular disease. Theranostics. 2021;11:3052–9. 10.7150/thno.54113 PMC7847684 33537073

[21] Li J, Jia Y-C, Ding Y-X, Bai J, Cao F, Li F. The crosstalk between ferroptosis and mitochondrial dynamic regulatory networks. Int J Biol Sci. 2023;19:2756–71. 10.7150/ijbs.83348 PMC10266069 37324946

[22] Reed A, Ichu T-A, Milosevich N, Melillo B, Schafroth MA, Otsuka Y, et al. LPCAT3 inhibitors remodel the polyunsaturated phospholipid content of human cells and protect from ferroptosis. ACS Chem Biol. 2022;17:1607–18. 10.1021/acschembio.2c00317 35658397

[23] Lin Z, Long F, Kang R, Klionsky DJ, Yang M, Tang D. The lipid basis of cell death and autophagy. Autophagy. 2024;20:469–88. 10.1080/15548627.2023.2259732 PMC10936693 37768124

[24] Tian Y, Jellinek MJ, Mehta K, Seok SM, Kuo SH, Lu W, et al. Membrane phospholipid remodeling modulates nonalcoholic steatohepatitis progression by regulating mitochondrial homeostasis. Hepatol Balt Md. 2024;79:882–97. 10.1097/HEP.0000000000000375 PMC10544743 36999536

[25] Kondreddy V, Banerjee R, Devi BLAP, Muralidharan K, Piramanayagam S. Inhibition of the MALT1-LPCAT3 axis protects cartilage degeneration and osteoarthritis. Cell Commun Signal. 2024;22:189. 10.1186/s12964-024-01547-4 PMC10960471 38519981

[26] Hu J, Deng Y, Ding T, Dong J, Liang Y, Lou B. Lpcat3 deficiency promotes palmitic acid-induced 3T3-L1 mature adipocyte inflammation through enhanced ROS generation. Acta Biochim Biophys Sin. 2022;55:117–30. 10.3724/abbs.2022161 PMC10157521 36331295

[27] Ding Y, Cui K, Han S, Hao T, Liu Y, Lai W, et al. Lysophosphatidylcholine acyltransferase 3 (LPCAT3) mediates palmitate-induced inflammation in macrophages of large yellow croaker (Larimichthys crocea). Fish Shellfish Immunol. 2022;126:12–20. 10.1016/j.fsi.2022.05.003 35526799

[28] Wang J, Zhang M, Ding Y, Lin Y, Xue Y, Wang X, et al. Coronaviral main protease induces LPCAT3 cleavage and endoplasmic reticulum (ER) stress. Viruses. 2023;15:1696. 10.3390/v15081696 PMC10457833 37632038

[29] Shi S, Zhang H, Jiang P, Zhou Y, Zhu Y, Feng T, et al. Inhibition of LPCAT3 exacerbates endoplasmic reticulum stress and HBV replication. Int Immunopharmacol. 2024;143:113337. 10.1016/j.intimp.2024.113337 39423656

[30] Feng B, Guo H-Y, Ning Y, Zhao Y-Y, Wang X, Cui R. LPCAT3 regulates the immune infiltration and prognosis of ccRCC patients by mediating ferroptosis and endoplasmic reticulum stress. Discov Oncol. 2025;16:574. 10.1007/s12672-025-02283-y PMC12009263 40253575

[31] Rong X, Albert CJ, Hong C, Duerr MA, Chamberlain BT, Tarling EJ, et al. LXRs regulate ER stress and inflammation through dynamic modulation of membrane phospholipid composition. Cell Metab. 2013;18:685–97. 10.1016/j.cmet.2013.10.002 PMC3889491 24206663

[32] Lu Y, Shao M, Xiang H, Wang J, Ji G, Wu T. Qinggan huoxue recipe alleviates alcoholic liver injury by suppressing endoplasmic reticulum stress through LXR-LPCAT3. Front Pharm. 2022;13:824185. 10.3389/fphar.2022.824185 PMC9009225 35431945

[33] Zhang L, Fang J, Tang Z, Luo Y. A bioinformatics perspective on the dysregulation of ferroptosis and ferroptosis-related immune cell infiltration in Alzheimer’s disease. Int J Med Sci. 2022;19:1888–902. 10.7150/ijms.76660 PMC9682502 36438927

[34] Thomas C, Jalil A, Magnani C, Ishibashi M, Queré R, Bourgeois T, et al. LPCAT3 deficiency in hematopoietic cells alters cholesterol and phospholipid homeostasis and promotes atherosclerosis. Atherosclerosis. 2018;275:409–18. 10.1016/j.atherosclerosis.2018.05.023 29866392

[35] Kleiboeker B, He A, Tan M, Lu D, Hu D, Liu X, et al. Adipose tissue peroxisomal lipid synthesis orchestrates obesity and insulin resistance through LXR-dependent lipogenesis. Mol Metab. 2024;82:101913. 10.1016/j.molmet.2024.101913 PMC10950804 38458567

[36] Kawamura S, Matsushita Y, Kurosaki S, Tange M, Fujiwara N, Hayata Y, et al. Inhibiting SCAP/SREBP exacerbates liver injury and carcinogenesis in murine nonalcoholic steatohepatitis. J Clin Invest. 2022;132:e151895. 10.1172/JCI151895 PMC9151706 35380992

[37] Sha W, Hu F, Xi Y, Chu Y, Bu S. Mechanism of ferroptosis and its role in type 2 diabetes mellitus. J Diab Res. 2021;2021:9999612. 10.1155/2021/9999612 PMC8257355 34258295

[38] Hishikawa D, Shindou H, Kobayashi S, Nakanishi H, Taguchi R, Shimizu T. Discovery of a lysophospholipid acyltransferase family essential for membrane asymmetry and diversity. Proc Natl Acad Sci. 2008;105:2830–5. 10.1073/pnas.0712245105 PMC2268545 18287005

[39] Zhang Q, Yao D, Rao B, Jian L, Chen Y, Hu K, et al. The structural basis for the phospholipid remodeling by lysophosphatidylcholine acyltransferase 3. Nat Commun. 2021;12:6869. 10.1038/s41467-021-27244-1 PMC8617236 34824256

[40] Kazachkov M, Chen Q, Wang L, Zou J. Substrate preferences of a lysophosphatidylcholine acyltransferase highlight its role in phospholipid remodeling. Lipids. 2008;43:895–902. 10.1007/s11745-008-3233-y 18781350

[41] Bousquet D, Guillot N. Laminar shear stress promotes accumulation of polyunsaturated fatty acid in aortic endothelial cells through upregulation of LPCAT3 enzyme activity. Biochim Biophys Acta Mol Cell Biol Lipids. 2025;1870:159638. 10.1016/j.bbalip.2025.159638 40414415

[42] Chen Z, Rand RP. The influence of cholesterol on phospholipid membrane curvature and bending elasticity. Biophys J. 1997;73:267–76. 10.1016/S0006-3495(97)78067-6 PMC1180928 9199791

[43] Gormal RS, Padmanabhan P, Kasula R, Bademosi AT, Coakley S, Giacomotto J, et al. Modular transient nanoclustering of activated β2-adrenergic receptors revealed by single-molecule tracking of conformation-specific nanobodies. Proc Natl Acad Sci USA. 2020;117:30476–87. 10.1073/pnas.2007443117 PMC7720173 33214152

[44] Momboisse F, Nardi G, Colin P, Hery M, Cordeiro N, Blachier S, et al. Tracking receptor motions at the plasma membrane reveals distinct effects of ligands on CCR5 dynamics depending on its dimerization status. eLife. 2022;11:e76281. 10.7554/eLife.76281 PMC9307273 35866628

[45] Boltz H-H, Sirbu A, Stelzer N, de Lanerolle P, Winkelmann S, Annibale P. The impact of membrane protein diffusion on GPCR signaling. Cells. 2022;11:1660. 10.3390/cells11101660 PMC9139411 35626696

[46] Kockelkoren G, Lauritsen L, Shuttle CG, Kazepidou E, Vonkova I, Zhang Y, et al. Molecular mechanism of GPCR spatial organization at the plasma membrane. Nat Chem Biol. 2024;20:142–50. 10.1038/s41589-023-01385-4 PMC10792125 37460675

[47] Shao G, Qian Y, Lu L, Liu Y, Wu T, Ji G, et al. Research progress in the role and mechanism of LPCAT3 in metabolic related diseases and cancer. J Cancer. 2022;13:2430–9. 10.7150/jca.71619 PMC9174858 35711841

[48] Mukherjee P, Chattopadhyay A, Fogelman AM. The role of the small intestine in modulating metabolism and inflammation in atherosclerosis and cancer. Curr Opin Lipido. 2019;30:383–7. 10.1097/MOL.0000000000000629 PMC6953609 31356236

[49] Sun D, Wang L, Wu Y, Yu Y, Yao Y, Yang H, et al. Lipid metabolism in ferroptosis: mechanistic insights and therapeutic potential. Front Immunol. 2025;16:1545339. 10.3389/fimmu.2025.1545339 PMC11932849 40134420

[50] Jiang H, Li Z, Huan C, Jiang X-C. Macrophage lysophosphatidylcholine acyltransferase 3 deficiency-mediated inflammation is not sufficient to induce atherosclerosis in a mouse model. Front Cardiovasc Med. 2018;5:192. 10.3389/fcvm.2018.00192 PMC6344406 30705887

[51] Liu N, Sun Q, Xu H, Yu X, Chen W, Wei H, et al. Hyperuricemia induces lipid disturbances mediated by LPCAT3 upregulation in the liver. FASEB J. 2020;34:13474–93. 10.1096/fj.202000950R 32780898

[52] Yu W, Wang J, Xiong Y, Liu J, Baranenko D, Cifuentes A, et al. Impact of imperata cylindrica polysaccharide on liver lipid metabolism disorders caused by hyperuricemia. Int J Biol Macromol. 2024;283:137592. 10.1016/j.ijbiomac.2024.137592 39557274

[53] Lin D, Zhang M, Luo C, Wei P, Cui K, Chen Z. Targeting ferroptosis attenuates inflammation, fibrosis, and mast cell activation in chronic prostatitis. J Immunol Res. 2022;2022:6833867. 10.1155/2022/6833867 PMC9232311 35755168

[54] Lin D, Gold A, Kaye S, Atkinson JR, Tol M, Sas A, et al. Arachidonic acid mobilization and peroxidation promote microglial dysfunction in Aβ pathology. J Neurosci. 2024;44:e0202242024. 10.1523/JNEUROSCI.0202-24.2024 PMC11293449 38866484

[55] Zhang T-C, Lin Y-C, Sun N-N, Liu S, Hu W-Z, Zhao Y, et al. Icariin, astragaloside a and puerarin mixture attenuates cognitive impairment in APP/PS1 mice via inhibition of ferroptosis-lipid peroxidation. Neurochem Int. 2024;175:105705. 10.1016/j.neuint.2024.105705 38412923

[56] Cash JG, Hui DY. Liver-specific overexpression of LPCAT3 reduces postprandial hyperglycemia and improves lipoprotein metabolic profile in mice. Nutr Diab. 2016;6:e206. 10.1038/nutd.2016.12 PMC4855257 27110687

[57] He M, Li Z, Tung VSK, Pan M, Han X, Evgrafov O, et al. Inhibiting phosphatidylcholine remodeling in adipose tissue increases insulin sensitivity. Diabetes. 2023;72:1547–59. 10.2337/db23-0317 PMC10588299 37625119

[58] Tian Y, Mehta K, Jellinek MJ, Sun H, Lu W, Shi R, et al. Hepatic phospholipid remodeling modulates insulin sensitivity and systemic metabolism. Adv Sci. 2023;10:e2300416. 10.1002/advs.202300416 PMC10288282 37088778

[59] Tian Y, Lu W, Shi R, McGuffee R, Lee R, Ford DA, et al. Targeting phospholipid remodeling pathway improves insulin resistance in diabetic mouse models. FASEB J. 2023;37:e23251. 10.1096/fj.202301122RR PMC10575708 37823674

[60] Ferrara PJ, Rong X, Maschek JA, Verkerke AR, Siripoksup P, Song H, et al. Lysophospholipid acylation modulates plasma membrane lipid organization and insulin sensitivity in skeletal muscle. J Clin Invest. 2021;131:e135963. 10.1172/JCI135963 PMC8262507 33591957

[61] Tol MJ, Shimanaka Y, Bedard AH, Sapia J, Cui L, Colaço-Gaspar M. et al. Dietary control of peripheral adipose storage capacity through membrane lipid remodelling. Nat Metab. 2025;7:1424–1442. 10.1038/s42255-025-01320-y 40579620

[62] Wang B, Rong X, Duerr MA, Hermanson DJ, Hedde PN, Wong JS, et al. Intestinal phospholipid remodeling is required for dietary-lipid uptake and survival on a high-fat diet. Cell Metab. 2016;23:492–504. 10.1016/j.cmet.2016.01.001 PMC4785086 26833026

[63] Wolfgang MJ. Remodeling glycerophospholipids affects obesity-related insulin signaling in skeletal muscle. J Clin Invest. 2021;131:e148176. 10.1172/JCI148176 PMC8262494 33855969

[64] Ke P, Bao X, Liu C, Zhou B, Huo M, Chen Y, et al. LPCAT3 is a potential prognostic biomarker and may be correlated with immune infiltration and ferroptosis in acute myeloid leukemia: a pan-cancer analysis. Transl Cancer Res. 2022;11:3491–505. 10.21037/tcr-22-985 PMC9641088 36388050

[65] Wei J, Nai GY, Dai Y, Huang XJ, Xiong MY, Yao XY, et al. Dipetidyl peptidase-4 and transferrin receptor serve as prognostic biomarkers for acute myeloid leukemia. Ann Transl Med. 2021;9:1381. 10.21037/atm-21-3368 PMC8506534 34733933

[66] Zhong L, Zheng J, Wang Z, Lin L, Cong Q, Qiao L. Metabolomics and proteomics reveal the inhibitory effect of Lactobacillus crispatus on cervical cancer. Talanta. 2025;281:126839. 10.1016/j.talanta.2024.126839 39265423

[67] Zhang Q, Zhou J, Zhai D, Jiang Q, Yang M, Zhou M. Gut microbiota regulates the ALK5/NOX1 axis by altering glutamine metabolism to inhibit ferroptosis of intrahepatic cholangiocarcinoma cells. Biochim Biophys Acta Mol Basis Dis. 2024;1870:167152. 10.1016/j.bbadis.2024.167152 38582012

[68] Wang B, Rong X, Palladino END, Wang J, Fogelman AM, Martín MG, et al. Phospholipid remodeling and cholesterol availability regulate intestinal stemness and tumorigenesis. Cell Stem Cell. 2018;22:206–20.e4. 10.1016/j.stem.2017.12.017 PMC5807072 29395055

[69] Mokhtarpour K, Razi S, Rezaei N. Ferroptosis as a promising targeted therapy for triple negative breast cancer. Breast Cancer Res Treat. 2024;207:497–513. 10.1007/s10549-024-07387-7 38874688

[70] Freitas-Cortez MA, Masrorpour F, Jiang H, Mahmud I, Lu Y, Huang A, et al. Cancer cells avoid ferroptosis induced by immune cells via fatty acid binding proteins. Mol Cancer. 2025;24:40. 10.1186/s12943-024-02198-2 PMC11789333 39901247

[71] Shi Y, Wang Y, Dong H, Niu K, Zhang W, Feng K, et al. Crosstalk of ferroptosis regulators and tumor immunity in pancreatic adenocarcinoma: novel perspective to mRNA vaccines and personalized immunotherapy. Apoptosis. 2023;28:1423–35. 10.1007/s10495-023-01868-8 PMC10425492 37369808

[72] Gavini CK, Bookout AL, Bonomo R, Gautron L, Lee S, Mansuy-Aubert V. Liver X receptors protect dorsal root ganglia from obesity-induced endoplasmic reticulum stress and mechanical allodynia. Cell Rep. 2018;25:271–7.e4. 10.1016/j.celrep.2018.09.046 PMC7732131 30304667

[73] Bousquet D, Nader E, Connes P, Guillot N. Liver X receptor agonist upregulates LPCAT3 in human aortic endothelial cells. Front Physiol. 2024;15:1388404. 10.3389/fphys.2024.1388404 PMC11061552 38694208

[74] Lin Y, Sun Y, Hou W, Chen X, Zhou F, Xu Q, et al. FTO-mediated regulation of m6A methylation is closely related to apoptosis induced by repeated UV irradiation. J Dermatol Sci. 2024;114:124–32. 10.1016/j.jdermsci.2024.01.001 38749796

[75] Xu L, Wang Q-J, Nie M-X, Chen Z-F. Methyltransferase-like 14 promotes ferroptosis in sepsis-induced acute kidney injury via increasing the m6A methylation modification of LPCAT3. Mol Genet Genomics MGG. 2025;300:16. 10.1007/s00438-024-02219-1 39836248

[76] Habaxi K, Wang W, Taximaimaiti M, Wang L. Methylation regulation of LPCAT3 improves osteoarthritis by regulating ACSL4 to inhibit chondrocyte ferroptosis. Crit Rev Eukaryot Gene Expr. 2024;34:77–86. 10.1615/CritRevEukaryotGeneExpr.2023049244 38073444

[77] Dixon SJ, Lemberg KM, Lamprecht MR, Skouta R, Zaitsev EM, Gleason CE, et al. Ferroptosis: an iron-dependent form of nonapoptotic cell death. Cell. 2012;149:1060–72. 10.1016/j.cell.2012.03.042 PMC3367386 22632970

[78] Lee J-Y, Kim WK, Bae K-H, Lee SC, Lee E-W. Lipid metabolism and ferroptosis. Biology. 2021;10:184. 10.3390/biology10030184 PMC8000263 33801564

[79] Zhao J, Wang Q, Liu Z, Sun M, Zhou R, Fu ZF, et al. Z-ligustilide restricts rabies virus replication by inducing ferroptosis through the ACSL4-LPCAT3-POR pathway. Vet Microbiol. 2024;298:110260. 10.1016/j.vetmic.2024.110260 39316946

[80] Liu M, Zhou X, Li Y, Ma S, Pan L, Zhang X, et al. TIGAR alleviates oxidative stress in brain with extended ischemia via a pentose phosphate pathway-independent manner. Redox Biol. 2022;53:102323. 10.1016/j.redox.2022.102323 PMC9118922 35576689

[81] Liu M, Liu S, Lin Z, Chen X, Jiao Q, Du X, et al. Targeting the interplay between autophagy and the Nrf2 pathway in parkinson’s disease with potential therapeutic implications. Biomolecules. 2025;15:149. 10.3390/biom15010149 PMC11764135 39858542

[82] Parzych KR, Klionsky DJ. An overview of autophagy: morphology, mechanism, and regulation. Antioxid Redox Signal. 2014;20:460–73. 10.1089/ars.2013.5371 PMC3894687 23725295

[83] Lin H, Shen W, Luo B, Cao W, Qin X, Gao J, et al. Proteomic analysis of vibrio parahaemolyticus-stimulated pinctada martensii proteins for antimicrobial activity, potential mechanisms, and key components. Antibiotics. 2024;13:1100. 10.3390/antibiotics13111100 PMC11590882 39596793

[84] Li Q, Zhou X, Zhang X, Zhang C, Zhang SO. Nuclear receptor signaling regulates compartmentalized phosphatidylcholine remodeling to facilitate thermosensitive lipid droplet fusion. Nat Commun. 2025;16:3955. 10.1038/s41467-025-59256-6 PMC12034805 40289189

[85] Matsushima G, Yanase Y, Nakagawa T, Goda M, Ozawa K, Hosoi T. Endoplasmic reticulum stress and unfolded protein response in immune cell function. Front Immunol. 2025;16:1694102. 10.3389/fimmu.2025.1694102 PMC12592176 41208954

[86] Di Conza G, Tsai C-H, Gallart-Ayala H, Yu Y-R, Franco F, Zaffalon L, et al. Tumor-induced reshuffling of lipid composition on the endoplasmic reticulum membrane sustains macrophage survival and pro-tumorigenic activity. Nat Immunol. 2021;22:1403–15. 10.1038/s41590-021-01047-4 PMC7611917 34686867

[87] Lin LL, Lin AY, Knopf JL. Cytosolic phospholipase A2 is coupled to hormonally regulated release of arachidonic acid. Proc Natl Acad Sci USA. 1992;89:6147–51. 10.1073/pnas.89.13.6147 PMC402139 1631101

[88] Eto M, Shindou H, Koeberle A, Harayama T, Yanagida K, Shimizu T. Lysophosphatidylcholine acyltransferase 3 is the key enzyme for incorporating arachidonic acid into glycerophospholipids during adipocyte differentiation. Int J Mol Sci. 2012;13:16267–80. 10.3390/ijms131216267 PMC3546689 23208369

[89] Pérez-Chacón G, Astudillo AM, Ruipérez V, Balboa MA, Balsinde J. Signaling role for lysophosphatidylcholine acyltransferase 3 in receptor-regulated arachidonic acid reacylation reactions in human monocytes. J Immunol. 2010;184:1071–8. 10.4049/jimmunol.0902257 20018618

[90] Xiang H, Shao M, Lu Y, Wang J, Wu T, Ji G. Kaempferol alleviates steatosis and inflammation during early non-alcoholic steatohepatitis associated with liver X receptor α-lysophosphatidylcholine acyltransferase 3 signaling pathway. Front Pharm. 2021;12:690736. 10.3389/fphar.2021.690736 PMC8273916 34262459

[91] Alzheimer's Association. 2023 Alzheimer's disease facts and figures. Alzheimers Dement. 2023;19:1598–1695. 10.1002/alz.13016 36918389

[92] Jucker M, Walker LC. Alzheimer’s disease: from immunotherapy to immunoprevention. Cell. 2023;186:4260–70. 10.1016/j.cell.2023.08.021 PMC10578497 37729908

[93] Twarowski B, Herbet M. Inflammatory processes in Alzheimer’s disease-pathomechanism, diagnosis and treatment: a review. Int J Mol Sci. 2023;24:6518. 10.3390/ijms24076518 PMC10095343 37047492

[94] Rostagno AA. Pathogenesis of Alzheimer’s disease. Int J Mol Sci. 2022;24:107. 10.3390/ijms24010107 PMC9820480 36613544

[95] Kursun O, Karatas H, Bariskaner H, Ozturk S. Arachidonic acid metabolites in neurologic disorders. CNS Neurol Disord Drug Targets. 2022;21:150–9. 10.2174/1871527320666210512013648 33982658

[96] Sultana R, Perluigi M, Butterfield DA. Lipid peroxidation triggers neurodegeneration: a redox proteomics view into the alzheimer disease brain. Free Radic Biol Med. 2013;62:157–69. 10.1016/j.freeradbiomed.2012.09.027 PMC3573239 23044265

[97] Tönnies E, Trushina E. Oxidative stress, synaptic dysfunction, and Alzheimer's disease. J Alzheimers Dis. 2017;57:1105–1121. 10.3233/JAD-161088 PMC5409043 28059794

[98] Tuo Q-Z, Zhang S-T, Lei P. Mechanisms of neuronal cell death in ischemic stroke and their therapeutic implications. Med Res Rev. 2022;42:259–305. 10.1002/med.21817 33957000

[99] Jia C-L, Gou Y, Gao Y, Pei X, Jin X, Li B-L, et al. Rosmarinic acid liposomes suppress ferroptosis in ischemic brain via inhibition of TfR1 in BMECs. Phytomed Int J Phytother Phytopharm. 2024;132:155835. 10.1016/j.phymed.2024.155835 38968791

[100] Zhu Y, Xian X, Wang Z, Bi Y, Chen Q, Han X, et al. Research progress on the relationship between atherosclerosis and inflammation. Biomolecules. 2018;8:80. 10.3390/biom8030080 PMC6163673 30142970

[101] Tedgui A, Mallat Z. Cytokines in atherosclerosis: pathogenic and regulatory pathways. Physiol Rev. 2006;86:515–81. 10.1152/physrev.00024.2005 16601268

[102] He W, Gao Z, Liu S, Tan L, Wu Y, Liu J, et al. G protein-coupled estrogen receptor activation by bisphenol-A disrupts lipid metabolism and induces ferroptosis in the liver. Environ Pollut. 2023;334:122211. 10.1016/j.envpol.2023.122211 37454720

[103] Feng F, Li X, Wang W, Dou M, Li S, Jin X, et al. Matrine protects against experimental autoimmune encephalomyelitis through modulating microglial ferroptosis. Biochem Biophys Res Commun. 2024;735:150651. 10.1016/j.bbrc.2024.150651 39260333

[104] Matboli M, Ahmed MF, Hasanin AH, Mohamed RH, Eltantawy EH, Hamam GG, et al. Amlodipine and perindopril modulate miR-874-3p expression to regulate ferroptosis-linked LPCAT3/LACS4/GPX4 Axis and gut dysbiosis in metabolic-associated steatohepatitis. Int Immunopharmacol. 2025;164:115333. 10.1016/j.intimp.2025.115333 40782429

[105] Kabir I, Li Z, Bui HH, Kuo M-S, Gao G, Jiang X-C. Small intestine but not liver lysophosphatidylcholine acyltransferase 3 (Lpcat3) deficiency has a dominant effect on plasma lipid metabolism. J Biol Chem. 2016;291:7651–60. 10.1074/jbc.M115.697011 PMC4817191 26828064

[106] Pang L, Xiong Y, Feng Z, Li C, Fang B, Huang Q, et al. Integrative analysis of transcriptome and metabolome to illuminate the protective effects of didymin against acute hepatic injury. Mediators Inflamm. 2023;2023:6051946. 10.1155/2023/6051946 PMC9851790 36687218

[107] Yu MG, Gordin D, Fu J, Park K, Li Q, King GL. Protective factors and the pathogenesis of complications in diabetes. Endocr Rev. 2024;45:227–52. 10.1210/endrev/bnad030 PMC10911956 37638875

[108] Bielka W, Przezak A, Molęda P, Pius-Sadowska E, Machaliński B. Double diabetes-when type 1 diabetes meets type 2 diabetes: definition, pathogenesis and recognition. Cardiovasc Diabetol. 2024;23:62. 10.1186/s12933-024-02145-x PMC10859035 38341550

[109] Młynarska E, Czarnik W, Dzieża N, Jędraszak W, Majchrowicz G, Prusinowski F, et al. Type 2 diabetes mellitus: new pathogenetic mechanisms, treatment and the most important complications. Int J Mol Sci. 2025;26:1094. 10.3390/ijms26031094 PMC11817707 39940862

[110] Feng H, Liu Q, Deng Z, Li H, Zhang H, Song J, et al. Human umbilical cord mesenchymal stem cells ameliorate erectile dysfunction in rats with diabetes mellitus through the attenuation of ferroptosis. Stem Cell Res Ther. 2022;13:450. 10.1186/s13287-022-03147-w PMC9444126 36064453

[111] Perdomo CM, Cohen RV, Sumithran P, Clément K, Frühbeck G. Contemporary medical, device, and surgical therapies for obesity in adults. Lancet Lond Engl. 2023;401:1116–30. 10.1016/S0140-6736(22)02403-5 36774932

[112] Rinella ME, Neuschwander-Tetri BA, Siddiqui MS, Abdelmalek MF, Caldwell S, Barb D, et al. AASLD practice guidance on the clinical assessment and management of nonalcoholic fatty liver disease. Hepatology. 2023;77:1797. 10.1097/HEP.0000000000000323 PMC10735173 36727674

[113] Zhou J, Zhou F, Wang W, Zhang X-J, Ji Y-X, Zhang P, et al. Epidemiological features of NAFLD from 1999 to 2018 in China. Hepatology. 2020;71:1851–64. 10.1002/hep.31150 32012320

[114] Younossi ZM, Golabi P, Paik JM, Henry A, Van Dongen C, Henry L. The global epidemiology of nonalcoholic fatty liver disease (NAFLD) and nonalcoholic steatohepatitis (NASH): a systematic review. Hepatology. 2023;77:1335–47. 10.1097/HEP.0000000000000004 PMC10026948 36626630

[115] Bednarski T, Rahim M, Whittenbarger N, Olichwier AJ, Young J. 260-LB: the effect of LXR activation on liver metabolism and diet-induced steatohepatitis in mice. Diabetes. 2023;72:260-LB.

[116] Hanahan D, Weinberg RA. Hallmarks of cancer: the next generation. Cell. 2011;144:646–74. 10.1016/j.cell.2011.02.013 21376230

[117] Dawson MA, Kouzarides T. Cancer epigenetics: from mechanism to therapy. Cell. 2012;150:12–27. 10.1016/j.cell.2012.06.013 22770212

[118] Bai X, Kang J, Wei S, Wang Y, Liu Y, Yuan B, et al. A pH responsive nanocomposite for combination sonodynamic-immunotherapy with ferroptosis and calcium ion overload via SLC7A11/ACSL4/LPCAT3 pathway. Exploration. 2025;5:20240002. 10.1002/EXP.20240002 PMC11875445 40040833

[119] Wang L, Yu H, Wang D, Yin G, Chen S, Zhang X, et al. Diosgenin alleviates lipid accumulation in NAFLD through the pathways of ferroptosis defensive and executive system. J Nutr Biochem. 2025;140:109886. 10.1016/j.jnutbio.2025.109886 40023201

[120] Wang Y, Zhang H, Liu J, Lin R, Huang Y, Liu X, et al. Curcumin nanoparticles attenuate sepsis-induced myocardial injury by modulating the Nrf2/HO-1/SLC7A11/GPX4 and ACSL4/LPCAT3 pathways. Pathol Res Pract. 2026;280:156385. 10.1016/j.prp.2026.156385 41619537

